# The regulatory effect of TiO_2_ nanotubes loaded with graphene oxide on macrophage polarization in an inflammatory environment

**DOI:** 10.1186/s12903-024-04608-9

**Published:** 2024-07-20

**Authors:** Xu Cao, Bin Luo, Yanting Mu, Caiyun Wang, Ran Lu, Yao Yao, Su Chen

**Affiliations:** Laboratory of Biomaterials and Biomechanics, Beijing Key Laboratory of Tooth Regeneration and Function Reconstruction, Beijing Stomatological Hospital, Capital Medical University, Beijing, 100050 China

**Keywords:** Biomaterials, Nanotubes, Graphene oxide, Immune regulation, Macrophage polarization, Reactive oxygen species

## Abstract

**Background:**

Excessive inflammation is a major cause of implant failure. The surface morphology, hydrophilicity, and loading of biomaterials are major properties modulating anti-inflammatory macrophage activation. This paper investigates the regulatory effects of modifying the surface of Titanium dioxide nanotubes (TNTs) with graphene oxide (GO) on the polarization of mouse monocyte macrophages (RAW264.7).

**Methods:**

TNT was produced by the anodic oxidation of titanium. GO was subsequently electrodeposited on the TNT to obtain a TNT–GO composite. The samples were characterised through scanning electron microscopy (SEM), Raman spectroscopy, and X-ray diffraction. RAW264.7 cells were separately seeded onto the surface of three groups of samples: pure Ti, TNT, and TNT–GO. Under the condition of lipopolysaccharide stimulation, the influence of the sample surfaces on the gene expression profiles was investigated through RNA sequence analysis. In addition, cell spreading was observed through SEM, cell adhesion and proliferation were analysed using the CCK8 assay, and the expression of inflammation-related factors was investigated by ELISA and cellular immunofluorescence staining. The production of reactive oxygen species (ROS) in the RAW264.7 cells on the surface of the three groups was detected via immunofluorescence staining.

**Results:**

The CCK8 results indicated that the adhesion and proliferation of the RAW264.7 cells were reduced on the TNT and TNT–GO surfaces. ELISA results revealed significant differences in the pro-inflammatory factors tumour necrosis factor-α and interleukin-6 secretion among the three groups at 24 h (*p* < 0.05). The secretion of pro-inflammatory factors significantly reduced and the expression of anti-inflammatory factor IL-10 increased on the TNT and TNT–GO surfaces. The RNA sequencing, ELISA, and cell immunofluorescence staining test results suggested that the inflammatory response of M1 polarization was reduced and the M2 polarization of macrophages was induced on the TNT–GO surface, which may be attributed to the reduction in ROS production.

**Conclusions:**

Under lipopolysaccharide stimulation, the inflammatory response of the RAW264.7 cells was reduced and the M2 polarization of macrophages was promoted on the TNT–GO surface, which may be caused by the reduced ROS production. Consequently, the designed TNT–GO material is promising for implants owing to its excellent inflammation regulation ability.

**Supplementary Information:**

The online version contains supplementary material available at 10.1186/s12903-024-04608-9.

## Background

Although titanium (Ti) has been widely employed in dental and orthopaedic implants because of its good biocompatibility and excellent mechanical properties [1, 2], implants might fail owing to poor bone resorption and bacterial infections [3–5]. A particular problem is excessive inflammation caused by adverse immune reactions, which is a major cause of implant failure. Consequently, the development of innovative materials that can promote implant integration and tissue healing is of utmost importance [6]. Under appropriate preparation conditions, titanium dioxide nanomaterials have been proven to exhibit good biocompatibility and a wide range of biological functions, which can increase the strength of repair materials, inhibit the reproduction of oral pathogenic bacteria, and promote implant osseointegration [7–11]. Our group previously reported that titanium dioxide nanotubes (TNTs) can promote the adhesion, migration, and extracellular matrix synthesis of human gingival fibroblasts [12], in addition to inducing some limited anti-inflammatory responses [13].

Owing to the complex cellular and molecular mechanisms underlying the crosstalk between materials and immune cells, this study aimed to develop ideal biomaterials with a microscopic morphology that can immunoregulate host–biomaterial interfaces. As macrophages with high heterogeneity and plasticity play a key role in coordinating foreign body responses and regulating the immune system, the initial macrophage-mediated inflammatory response is a key component of this complex process [14]. Furthermore, macrophages play a decisive role in all stages of tissue healing and can secrete chemokines and cytokines to regulate the tissue repair and reconstruction processes [15–17]. Macrophage polarization is a process by which macrophages adopt different functional programmes in response to signals from their microenvironment. Macrophages exhibit two polarization states depending on the microenvironment. The M1 phenotype promotes inflammatory responses and the secretion of tumour necrosis factor-α (TNF-α) and interleukin-6 (IL-6), whereas the M2 phenotype promotes the secretion of anti-inflammatory factors represented by IL-4 and IL-10, which weakens the inflammatory response, promotes wound healing, and accelerates tissue repair [18–21].

The high plasticity of macrophages and their effective response to the microenvironment can thus help develop novel immune regulatory materials. Consequently, regulating the macrophage polarization status by modifying the surface of biomaterials has been gaining attention as a potential strategy to inhibit inflammatory responses. Therefore, this study focused on developing an implant material with an ideal morphology and properties capable of immunomodulatory activity, thereby reducing inflammation and accelerating tissue healing.

Recently, graphene oxide (GO) has been gaining significant attention in the biomedical field owing to its good hydrophilicity, dispensability, and biocompatibility [22–24]. Furthermore, its rough micro-folding structure can regulate the tension of the cytoskeleton and promote cell adhesion [25, 26]. The accumulation of π–π bonds in the GO structure can also facilitate proliferation and migration [27]. Therefore, GO is a promising coating material for medical implants.

Lipopolysaccharide (LPS) is a major component of the outer membrane of gram-negative bacteria and is recognised by pattern recognition receptors such as Toll-like receptor 4 (TLR4). LPS can bind to TLR4 on the surface of macrophage membranes, activate transcription factors, and release inflammatory mediators such as nitric oxide (NO), TNF-α, and IL-6, accelerating the progression of inflammatory diseases [28]. Many pathogenic bacteria in the oral cavity are gram-negative bacteria; hence, the LPS-induced macrophage inflammation model is commonly used in oral research [13, 28].

In this study, an electrodeposition method was used to modify the surface of TNT with GO to obtain a TNT–GO composite material. Mouse monocyte macrophages (RAW 264.7) were cultured in vitro on the surfaces of pure Ti sheets, TNT, and TNT–GO. To investigate the ability of each surface to inhibit the inflammatory responses and regulate macrophage polarization, the RAW 264.7 cells under LPS stimulation were observed and evaluated on the surface of each group. Gene ontology was used to elucidate the possible mechanisms.

## Materials and methods

Commercially pure Ti sheets (99.99%; Cuibolin Nonferrous Metal Industry Co., Ltd., Beijing, China) were used in this study. The Ti sheets (10 × 10 × 0.2 mm) were ultrasonically washed successively with acetone (Eastern Fine Chemicals Co., Ltd., Beijing, China), anhydrous ethanol (Eastern Fine Chemicals Co., Ltd., Beijing, China), and deionised water for 10 min. They were then naturally dried and stored at room temperature to obtain clean Ti samples. The TNT array was obtained by anodisation (voltage 50 V, 15 min) followed by annealing at 550 °C for 2 h. GO was loaded onto the surface of the TNT by electrodeposition by using the TNT material as the positive electrode and a platinum sheet as the negative electrode. GO flakes (0.1 mg/mL, 100,681, XFNANO, Jiangsu, China) that were dispersed by ultrasonication for 2 h in advance were then loaded onto the TNT surface at a voltage of 50 V for 5 min to obtain the TNT–GO sample.

### Sample characterisation

The surface morphology of the materials was observed by scanning electron microscopy (SEM, S4800, Hitachi, Ltd., Tokyo, Japan). The reverse sides of the three groups (Ti, TNT, and TNT–GO) were coated with a conductive adhesive, marked, and pasted on the sample disk in sequence. The accelerated voltage was 10 kV, and the surface morphology of the samples was observed at a magnification of 50,000×. Raman spectra (1000–3000 cm^−1^, 532 nm line excitation source) were recorded using a LabRAM HR800 spectrometer (LabRAM HR800, HORIBA, USA) to determine the sample structure. The surface roughness of the samples was evaluated through atomic force microscopy (AFM; Nanoscope V, Veeco, Plainview, NY, USA). Three samples were randomly selected for each group of specimens. Three regions with the dimensions of 5 × 5 µm^2^ were randomly selected for each sample. The contact angles of the samples were determined using an optical contact-angle-measuring device (Model OCA15pro, Dataphysics Co., Ltd., Germany) to assess the hydrophilicity of the samples. The room temperature was 20 °C, and the relative humidity was kept below 21%. The Ti, TNT, and TNT–GO samples were placed on the loading platform, and 1 µL droplets of deionised water were suspended on the surfaces of different specimens using a microsampler. Three specimens were randomly selected in each group, and three points were randomly selected for each specimen for measurement. Photographs were captured at the same time point to analyse the water contact angles on the surfaces of different specimens. X-ray diffraction (XRD, TTRAX III, Rigaku Co., Tokyo, Japan) was performed to analyse the crystal phases of the samples with the dimensions of 10 × 10 × 0.2 mm, and the 2θ angle was 20°–80°.

### Cell culture

The commercial RAW264.7 cells, obtained from China Cell Line Resource Infrastructure (China Infrastructure of Cell Line Resource, Beijing, China), were cultured under standard culture conditions (37 °C, 5% CO_2_) in Dulbecco’s modified Eagle’s medium supplemented with 10% foetal bovine serum and 1% penicillin/streptomycin. To create pro-inflammatory conditions, all experiments were performed using a cell growth medium containing *Escherichia coli* O127:B8 LPS (1 µg/mL, L4516, Sigma, St. Louis, MO, USA) [13, 28]. All reagents used in this experiment were provided by Thermo Fisher Scientific (MA, USA).

### RNA sequence analysis (RNA-Seq)

The RAW 264.7 cells were seeded onto the samples in 24-well plates at a density of 2 × 10^5^ cells per well, and the total amount of RNA was extracted after 48 h using TRIzol kits (Invitrogen, Carlsbad, CA, USA). A NanoDrop 2000 spectrophotometer (Thermo Fisher Scientific, CA, USA) was used to evaluate RNA purity and quantity. An Agilent 2100 Bioanalyzer (Agilent Technologies, CA, USA) was utilised to evaluate the integrity of RNA. Based on the instructions provided by the manufacturer, libraries were prepared using the VAHTS Universal V6 RNA-seq Library Prep Kit. The transcriptome was sequenced and analysed by OE Biotech Co., Ltd. (Shanghai, China). The quality of the raw sequencing data was assessed using the FastQC software. The FPKM of each gene was calculated, and the read counts of each gene were obtained by HTSeq-count. To assess biological duplication, principal component analysis was performed using R (version 3.2.0), setting Q < 0.05, fold change > 2, or fold change < 0.5 as the thresholds of the significantly differentially expressed genes (DEGs). The gene ontology and Kyoto Encyclopedia of Genes and Genomes (KEGG) pathway enrichment analyses were performed using R (version 3.2.0).

### Morphology of macrophages

The RAW 264.7 cells were seeded onto the specimens at a density of 1 × 10^4^ cells per well in 24-well plates. Then, the cells were immediately stimulated by LPS (1 µg/mL) and cultured for 4–24 h, followed by fixing, dehydration, drying, and coating with gold. Finally, the morphology of the samples was observed through SEM (magnification: 2000×).

### Live/dead viability

The RAW 264.7 cells were seeded on the different samples at a density of 4 × 10^4^ cells per well in 24-well plates. After culturing macrophages on the surface of each group with LPS (1 µg/mL) for 24–48 h, diluted calcein–acetoxymethyl ester (250 µL) was added. After incubation for 30 min, a diluted propidium iodide solution (250 µL) was added. After another 30 min, the cells were observed using a confocal laser microscope (magnification: 100×).

### Cell adhesion and proliferation

The RAW 264.7 cells were seeded on the surface of each group at a density of 5 × 10^4^ cells per well in 24-well plates. The cells were cultured for 4, 24, or 48 h under LPS (1 µg/mL) stimulation. The cell counting kit-8 (CCK-8, Dojindo, Japan) was added 2 h before measuring the absorbance of the sample at 450 nm using a multifunctional enzyme marker.

### Secretion of cytokines

The RAW 264.7 cells were seeded onto the different samples at a density of 1 × 10^5^ cells per well in 24-well plates. Then, the cells were incubated under LPS (1 µg/mL) stimulation for 24–48 h. The supernatant on the surface of each group was collected and centrifuged at 6000 rpm for 10 min. The concentrations of TNF-α, IL-6, and IL-10 in the supernatant were determined by the enzyme-linked immunosorbent assay kit (ELISA, Abclonal, Wuhan, China) according to the instructions. Standard products were configured according to the instructions, and unknown samples were detected. Absorbance was measured at a wavelength of 450 nm with a multifunctional enzyme label instrument, and standard curves were plotted. Unknown sample protein concentrations were calculated according to the instructions.

### Cellular immunofluorescence of macrophage-polarization-related proteins

The RAW 264.7 cells were seeded onto the different samples at a density of 5 × 10^4^ cells per well in 24-well plates. The cells were incubated with LPS (1 µg/mL) for 24–48 h and then fixed with paraformaldehyde (4%) for 15 min. After sealing the sample with goat serum (10%), pro-inflammatory and anti-inflammatory biomarkers iNOS, CD80, ARG, and CD163 (all from, Abclonal, Wuhan, China) were labelled in green and combined with 4′,6-diamidino-2-phenylindole (DAPI, ZSBG-BIO, Beijing, China), which was used to stain the nucleus.

### Reactive oxygen species (ROS) production detection

The RAW 264.7 cells were seeded onto the different samples at a density of 4 × 10^4^ cells per well in 24-well plates. After incubating the RAW 264.7 cells with LPS (1 µg/mL) for 24–48 h, diacetyl dichlorofluorescein (10 µM/L, Beyotime, Shanghai, China) was added; the mixture was incubated at 37 °C for 30 min. The cells of the positive control group were stimulated with active oxygen species (Rosup, Beyotime, Shanghai, China) for 30 min at an excitation wavelength of 488 nm. The fluorescence intensity was detected at an emission wavelength of 525 nm, and the field of view was randomly selected for photography and quantitative analysis.

### Statistical analysis

The SPSS software (version 26.0; IBM Corp., USA) was used to perform the one-way analysis of variance (ANOVA). All data were expressed as mean ± standard deviation. Statistical significance was set to *p* < 0.05.

## Results

### Characteristics

**Fig. 1:**
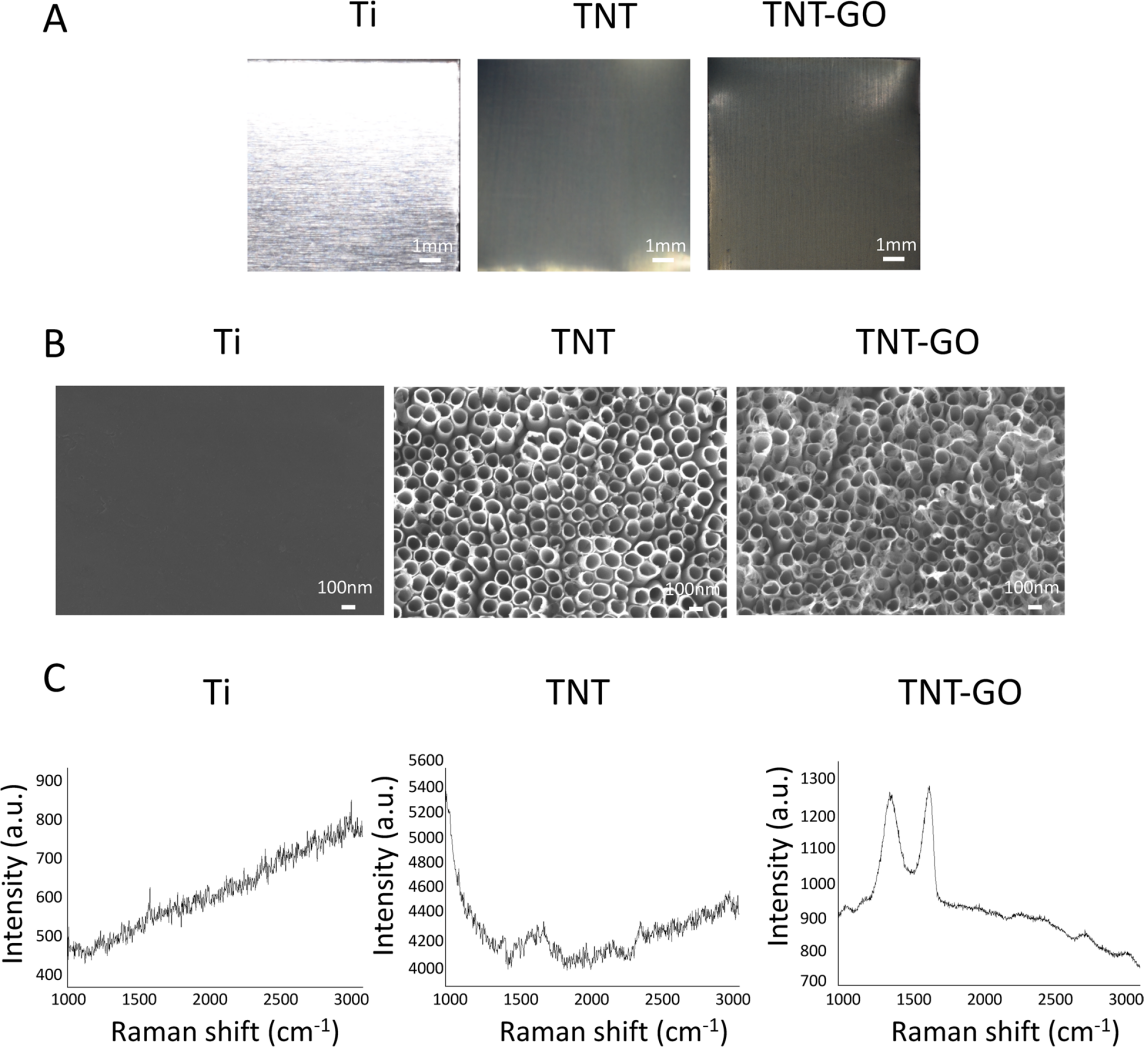
Surface characterisation of the Ti, TNT, and TNT–GO samples. **A** Photographs, **B** SEM images (magnification: 50,000×), and **C** Raman spectra of the samples

Visually examining the samples (Fig. 1A) revealed that the Ti surface was metallic; the TNT appeared light yellow and TNT–GO was yellowish brown. The SEM results (Fig. 1B) revealed that the Ti surface was smooth with a few scratches, whereas an array of TNT with diameters of ∼100 nm was observed in the TNT group. Moreover, a GO film deposited on the TNT was observed in the SEM image of TNT–GO. The Raman spectrum of TNT–GO (Fig. 1C) exhibited the two characteristic peaks of GO at 1350 and 1580 cm^−1^, confirming the successful loading of GO onto the TNT surface.

**Fig. 2:**
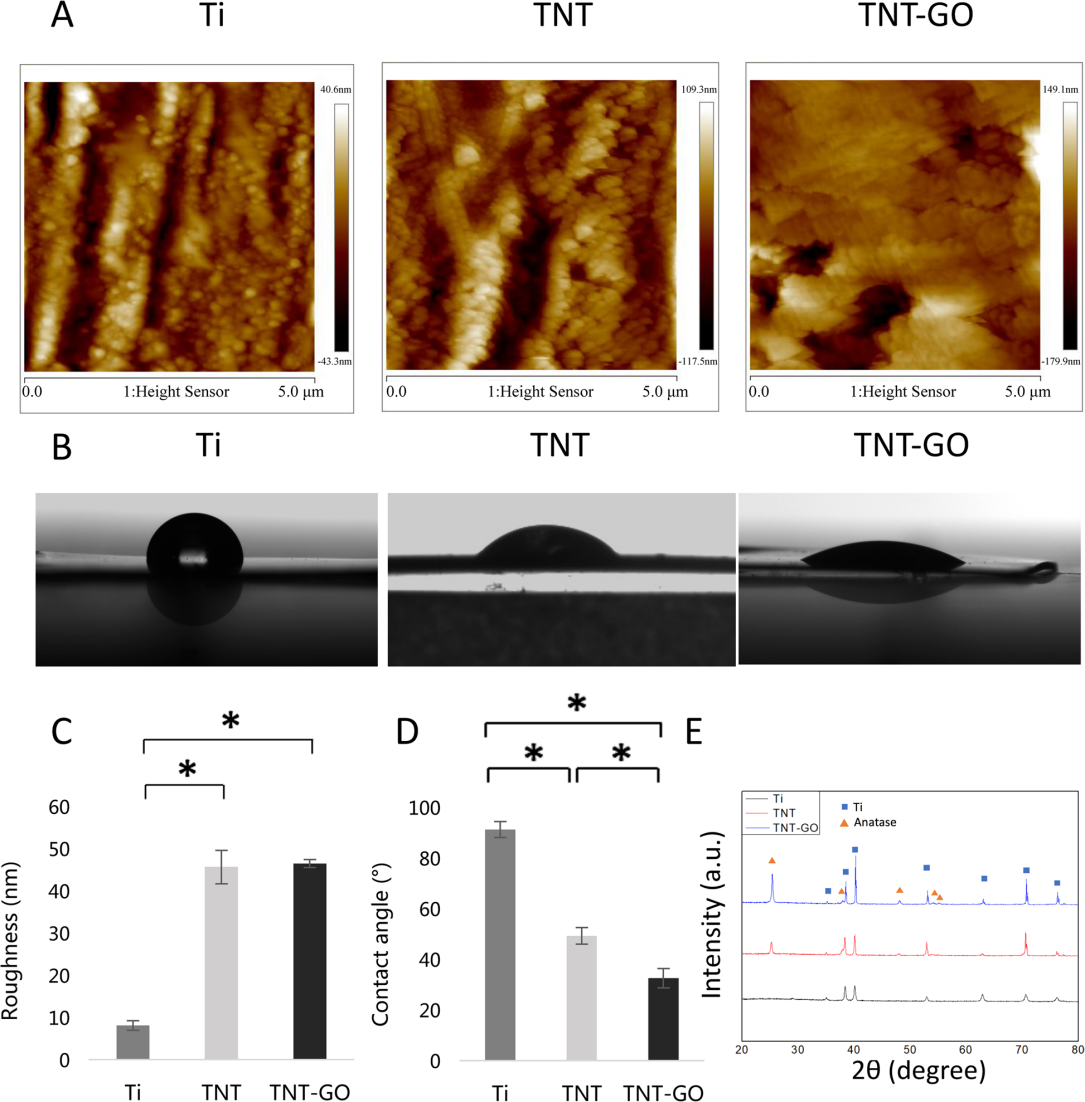
Surface analysis of the Ti, TNT, and TNT–GO samples. **A** Reconstructed AFM topographical images of the samples, **B** water contact angles, **C** surface roughness, **D** water contact angle, and **E** XRD patterns. *p < 0.05

The roughnesses of the Ti, TNT, and TNT–GO surfaces (Fig. 2A and C) were approximately 8.0 ± 1.1, 45.6 ± 4, and 46.5 ± 1.0 nm, respectively, while the corresponding water contact angles were 91.2° ± 3.2°, 49.2° ± 3.3°, and 32.5° ± 3.8° (Fig. 2B and D). In the XRD pattern of Ti, only the diffraction peak of Ti was observed (Fig. 2E); in contrast, the characteristic diffraction peaks of anatase-phase TiO_2_ were observed in that of TNT, in addition to the Ti diffraction peak. Moreover, the XRD patterns indicated that the crystal structure of TNT–GO was identical to that of TNT.

### RNA-Seq analysis

**Fig. 3:**
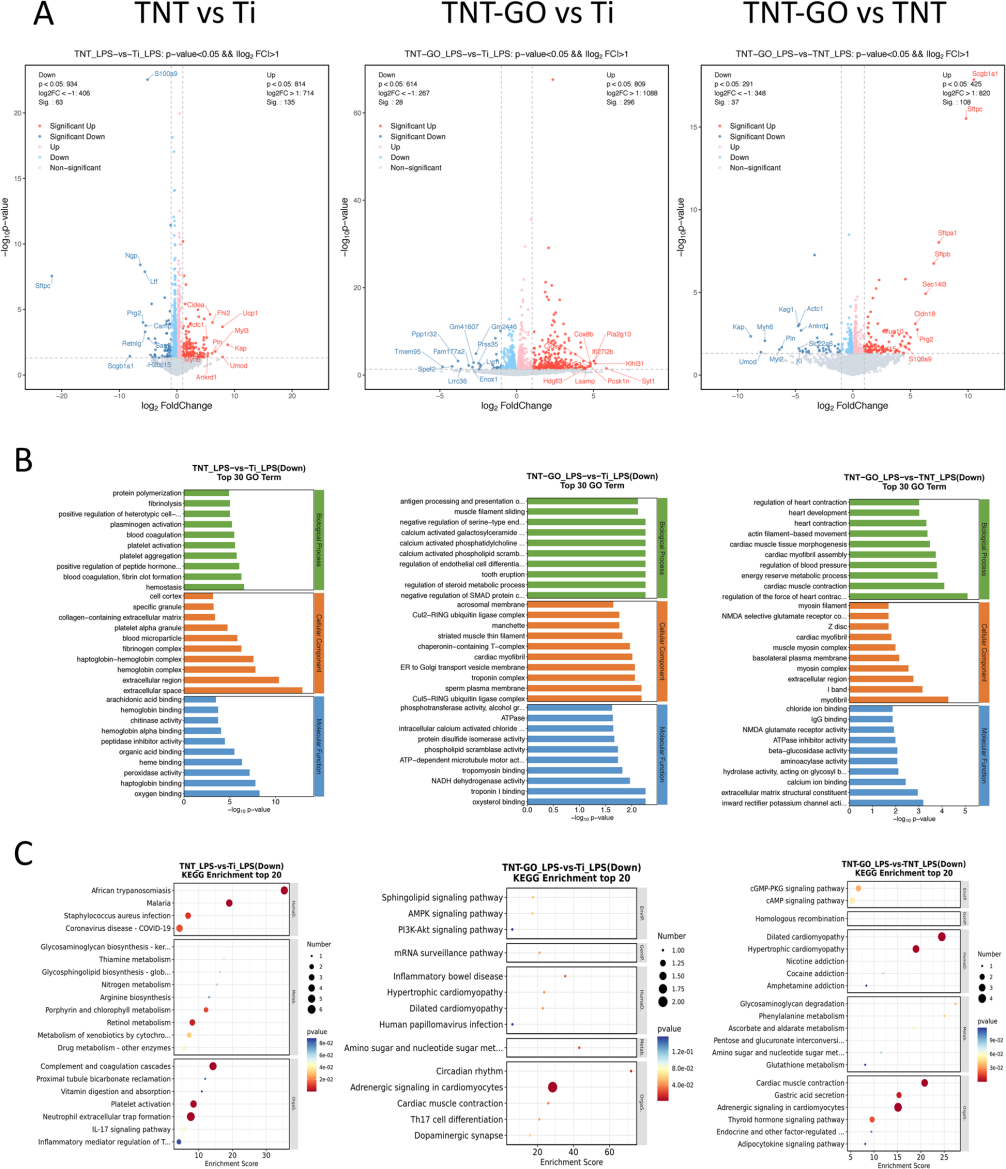
Results of RNA-Seq analysis of the RAW 264.7 cells on the Ti, TNT, and TNT–GO samples under LPS stimulation. **A** Volcano map showing DEGs in pairwise comparisons. The upregulated, downregulated, and unchanged genes are shown as red, blue, and grey dots, respectively; **B** gene ontology enrichment; and **C** KEGG enrichment of Ti, TNT, and TNT–GO

The results of the RNA-Seq analysis are shown in Fig. 3. The sequencing yielded approximately 65.2 Gb of raw data (142,547,120 sequences for Ti; 144,482,844 sequences of TNT; and 147,846,024 sequences of TNT–GO). In total, 198 DEGs were identified by comparing the TNT and Ti groups (Additional file 1), whereas the number of DEGs between TNT–GO and Ti was 324 and that between TNT–GO and TNT was 145 (Fig. 3A, Additional file 2 and 3).

Furthermore, the gene ontology analysis was performed to assess the functional changes in the genomic data (Fig. 3B and C, Additional file 4–9). The pairwise comparisons of TNT with Ti and TNT–GO with Ti revealed that the gene ontological terms were related to inflammatory processes, such as inflammation, oxygen binding, NADH dehydrogenase activity, and chemokine signals. By manually scanning the genetic data related to the inflammatory response and immune system processes, genes related to the inflammatory response and chemokine signalling pathways were found to be significantly downregulated in the TNT and TNT–GO groups, whereas the NADH dehydrogenase and NO activities were significantly reduced.

### Cell morphology

**Fig. 4:**
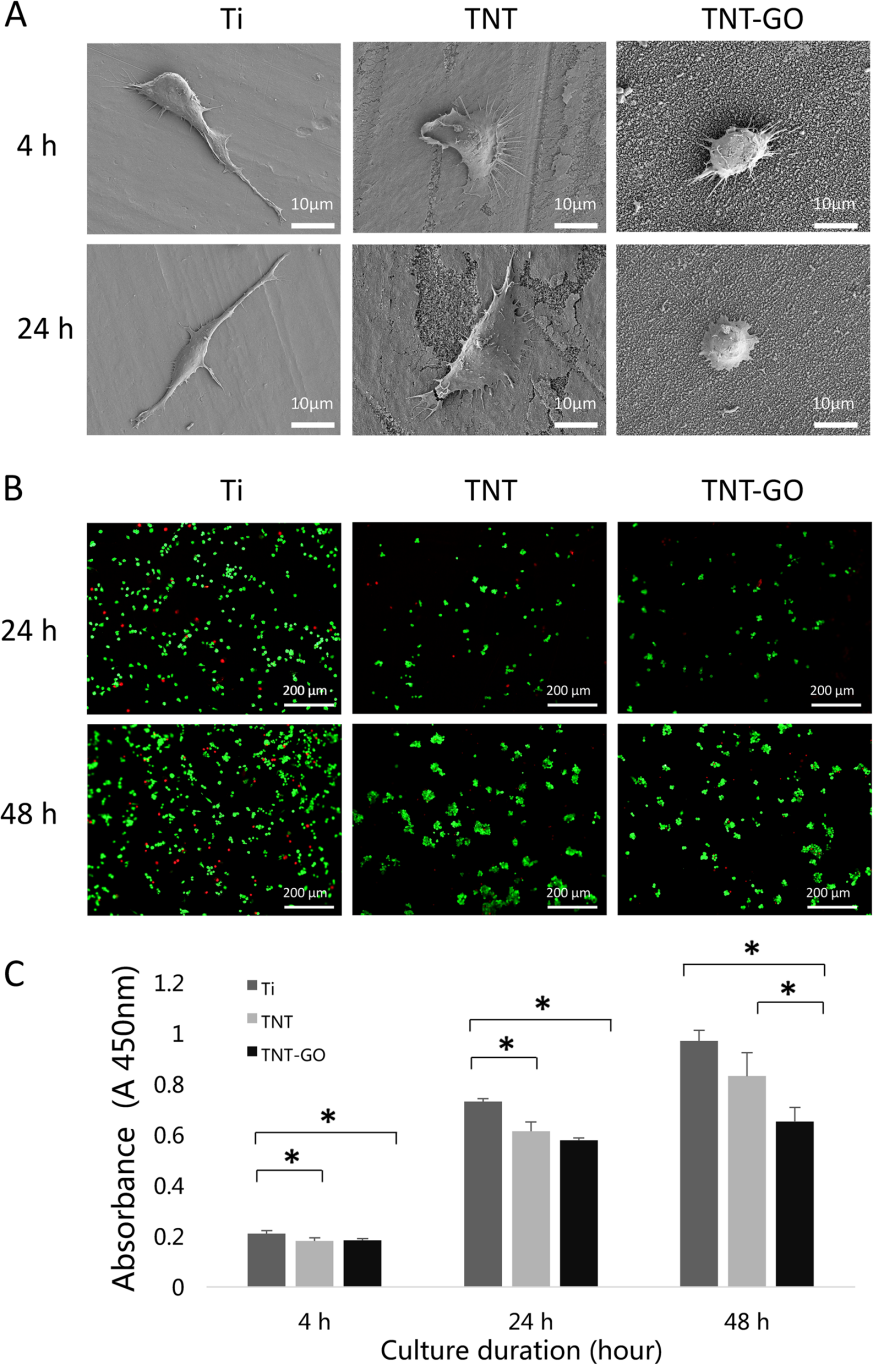
**A** SEM images of the RAW 264.7 cells on the different surfaces under LPS stimulation (magnification: 2000×). **B** Live/dead viability of the RAW 264.7 cells at 24 and 48 h under LPS stimulation (magnification 100×). **C** Cell adhesion and proliferation of the RAW 264.7 cells on the samples after 4, 24, and 48 h under LPS stimulation. *p < 0.05

The SEM images of the RAW 264.7 cells on the different surfaces under LPS stimulation (Fig. 4A) revealed that the cells in the Ti group were polygonal and had significantly elongated pseudopods, which are characteristic of M1-type macrophages. By contrast, the cells in the TNT group were semi-circular with small pseudopods, and the cells in the TNT–GO group were round or almost round.

### Fluorescent staining of living and dead cells

The fluorescent staining (Fig. 4B) results revealed that the number of macrophages on the surface of each group increased over time, with the green- and red-fluorescent dyes representing living and dead cells, respectively. At each time point, the surface of the Ti group exhibited the greatest number of the RAW 264.7 cells, proliferation was obvious, and an elongated cell morphology was observed. The proliferation of the macrophages and the number of dead cells in the TNT and TNT–GO groups were lower than those in the Ti group. In both these groups, the cells were circular.

### Cell adhesion and proliferation

The adhesion and proliferation of the RAW 264.7 cells (Fig. 4C) on the samples were also assessed, revealing that proliferation was inhibited in both the TNT and TNT–GO groups at 4 and 24 h, even under LPS stimulation. After 48 h, the TNT–GO group more effectively inhibited the proliferation of inflammatory cells than the TNT group.

### ELISA

**Fig. 5:**
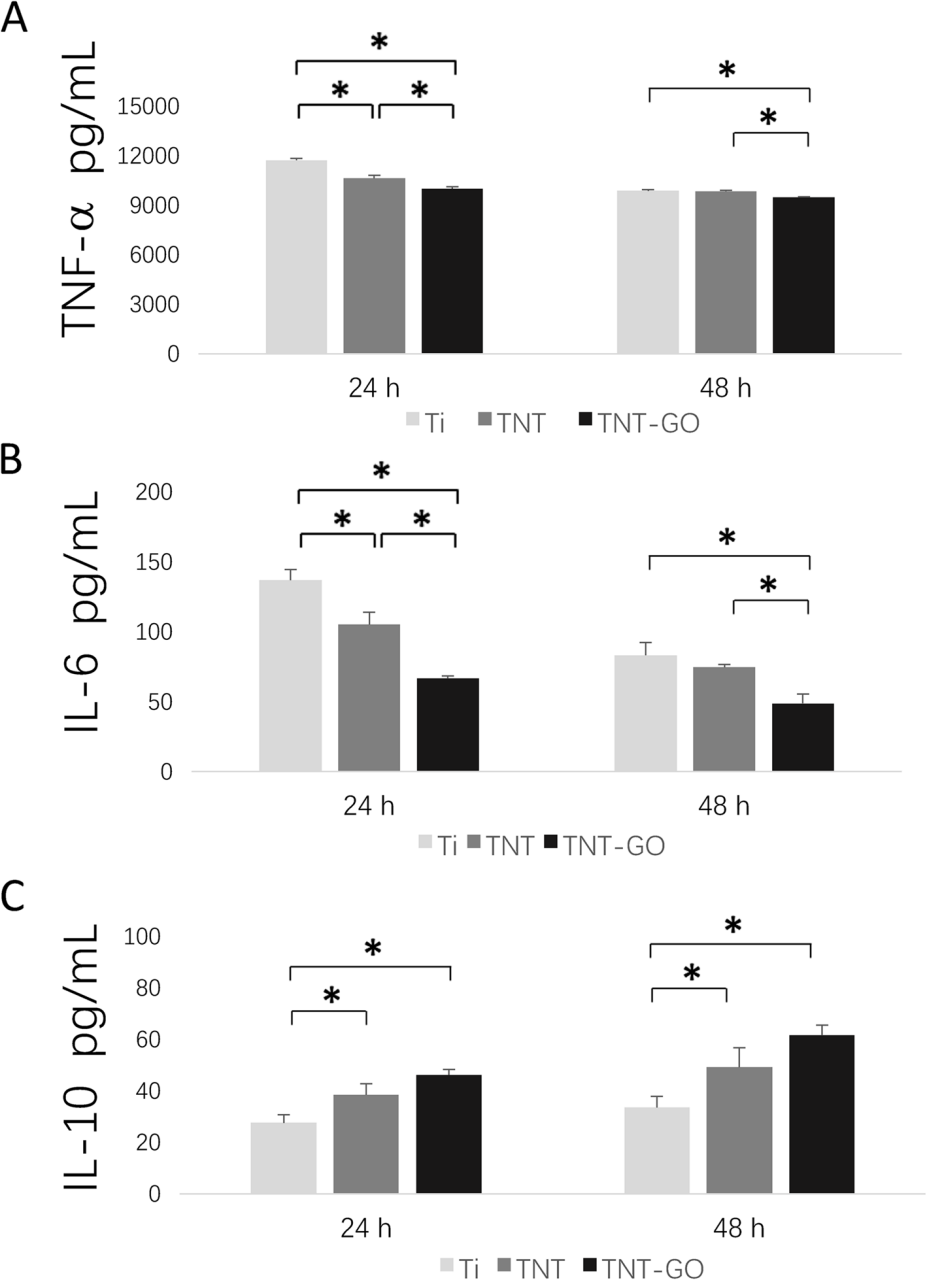
Pro- and anti-inflammatory cytokine levels under LPS stimulation at 24 and 48 h. **A** TNF-α, **B** IL-6, and **C** IL-10. *p < 0.05

The ELISA results (Fig. 5) revealed a large amount of TNF-α in the macrophages cultured on all surfaces owing to LPS stimulation. However, the secretion of M1-related cytokines TNF-α and IL-6 from the macrophages in the TNT–GO group was significantly low and that of M2-related factor (IL-10) was significantly high.

### Immunofluorescence staining

**Fig. 6:**
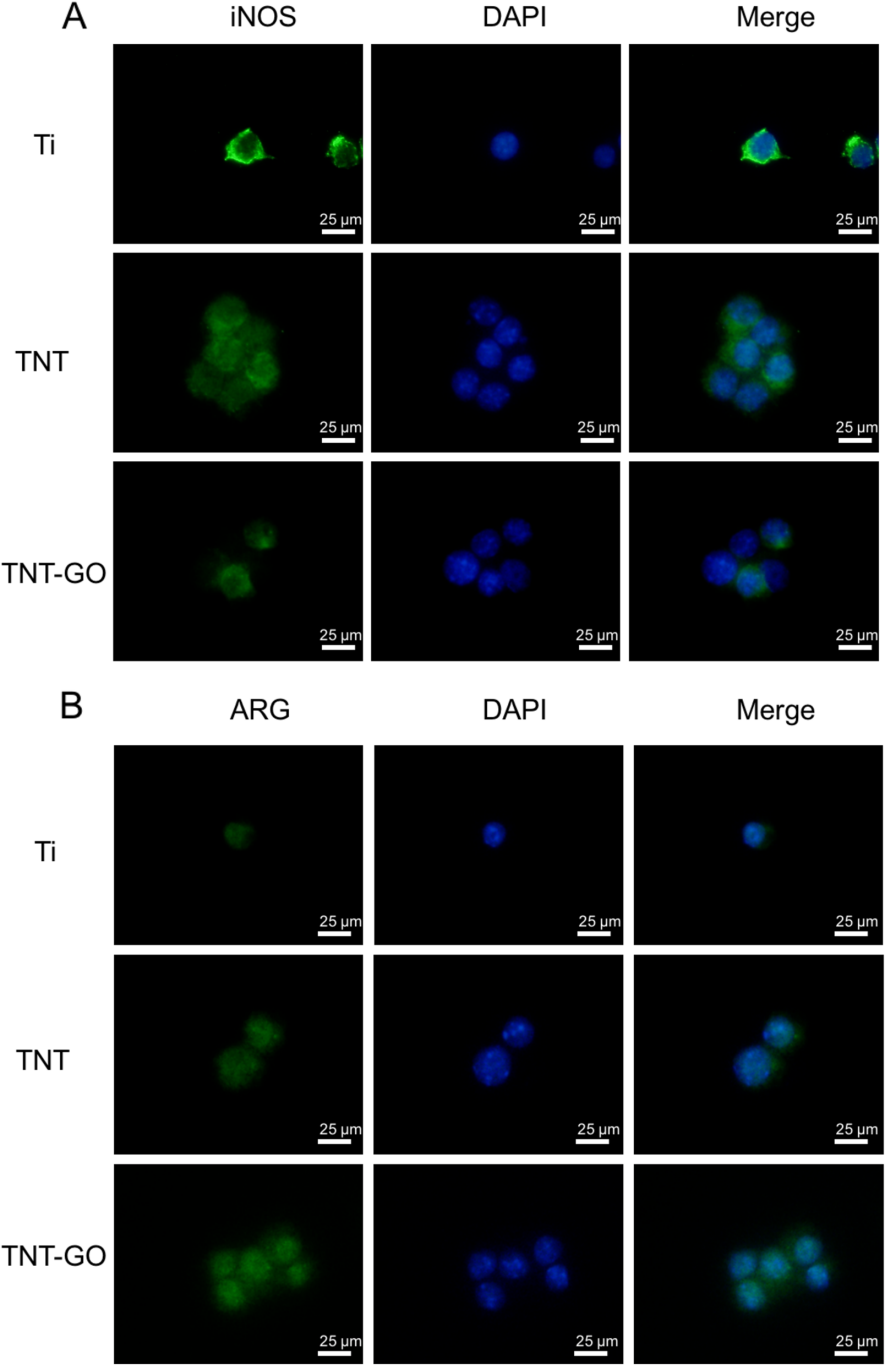
Immunofluorescence staining of the macrophages under LPS stimulation. **A** iNOS was labelled green, and nuclei were stained with DAPI. **B** ARG was labelled green, and nuclei were stained with DAPI. ‘Merge’ represents the merged images of iNOS or ARG and nuclei

**Fig. 7:**
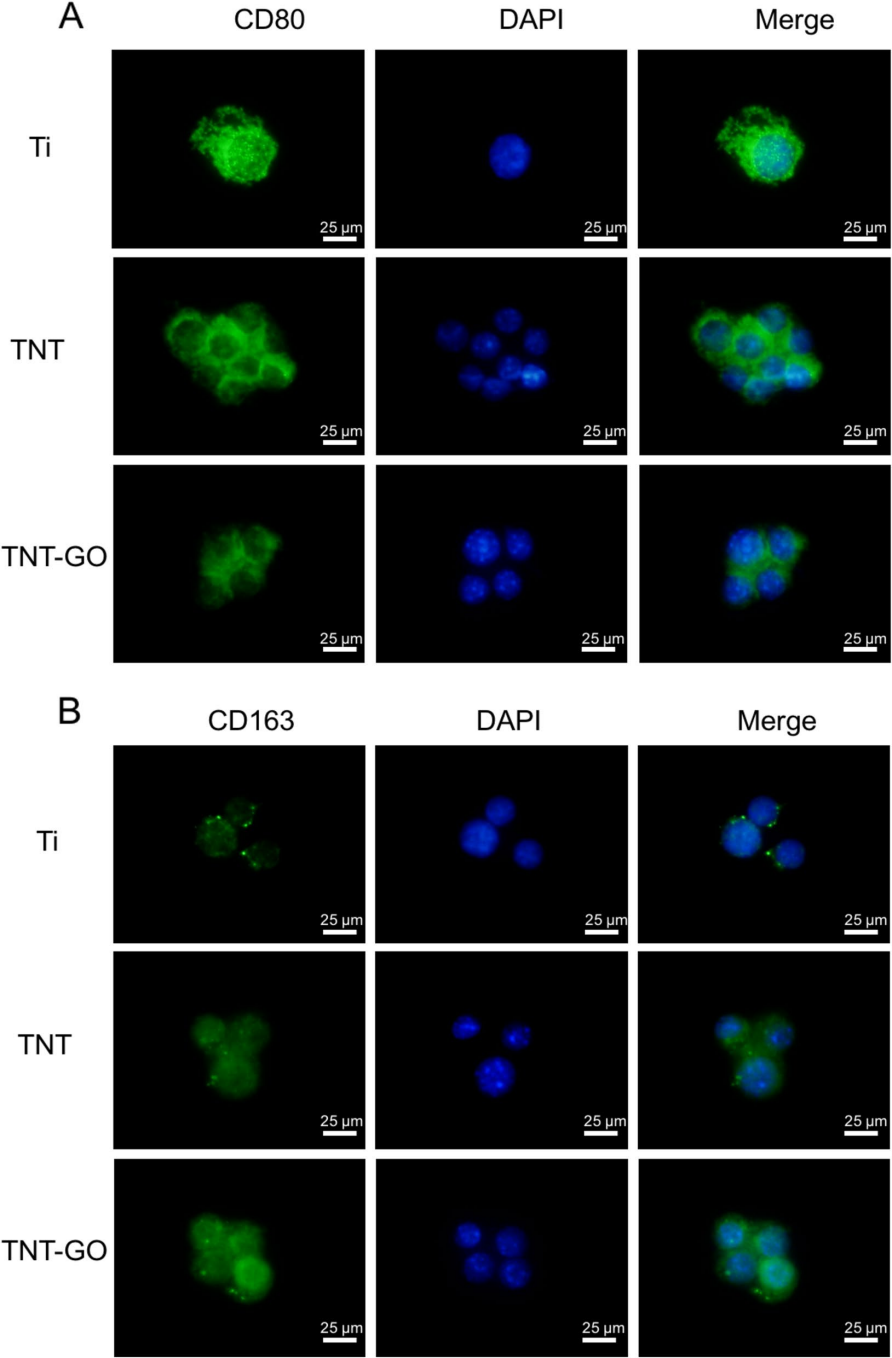
Immunofluorescence staining of the macrophages under LPS stimulation. **A** CD80 was labelled green, and nuclei were stained with DAPI. **B** CD163 was labelled green, and nuclei were stained with DAPI. ‘Merge’ represents the merged images of CD80 or CD163 and nuclei

**Fig. 8:**
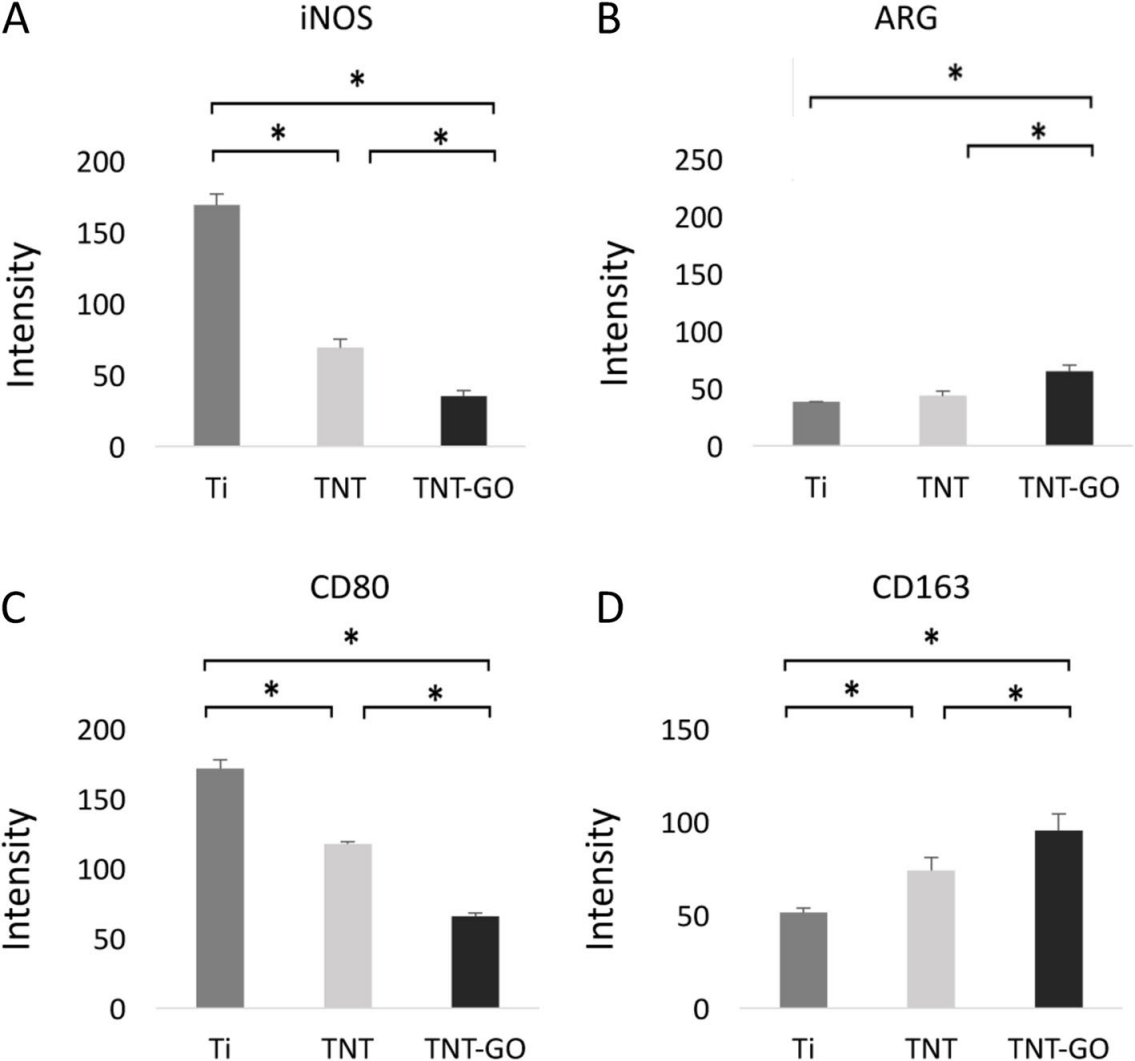
Results of quantitative analysis of fluorescent staining. **A** iNOS, **B** ARG, **C** CD80, and **D** CD163. *p < 0.05

The polarization-related protein expression results of the RAW 264.7 cells are shown in Figs. 6 and 7, and the fluorescence intensities were quantitatively analysed (Fig. 8). The expressions of the M1-related proteins, iNOS and CD80, in the TNT–GO group were significantly lower than those in the other two groups, and there were significant differences between the three groups. The expressions of the M2-related proteins, ARG and CD163, were significantly higher in the TNT–GO group.

### ROS generation

**Fig. 9:**
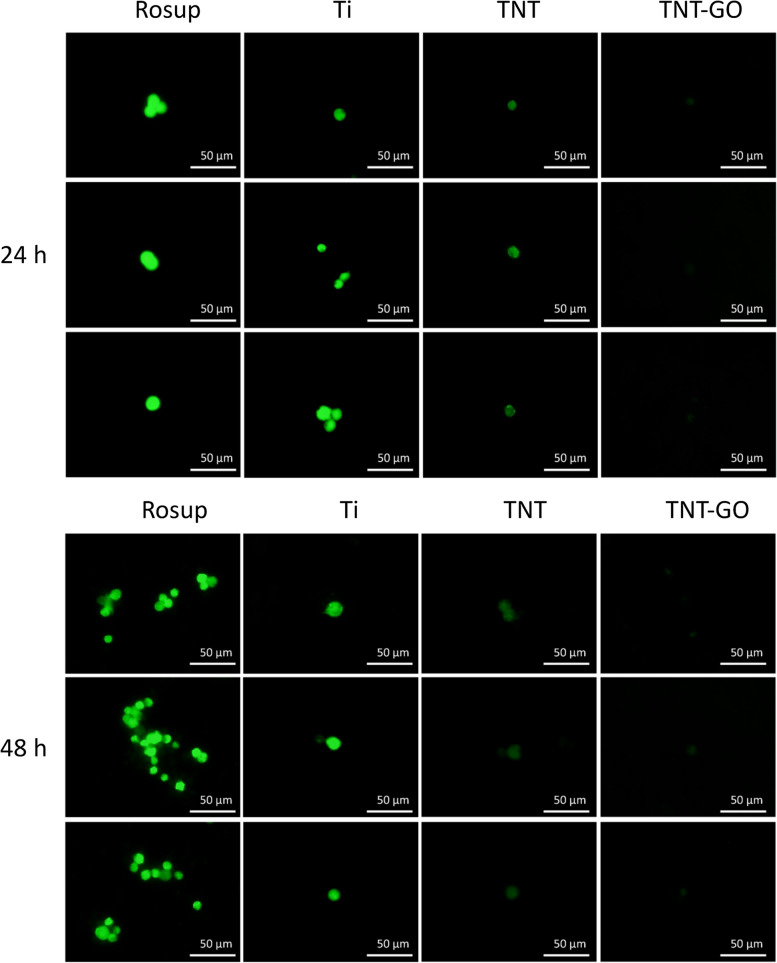
ROS production under LPS stimulation at 24 and 48 h

**Fig. 10:**
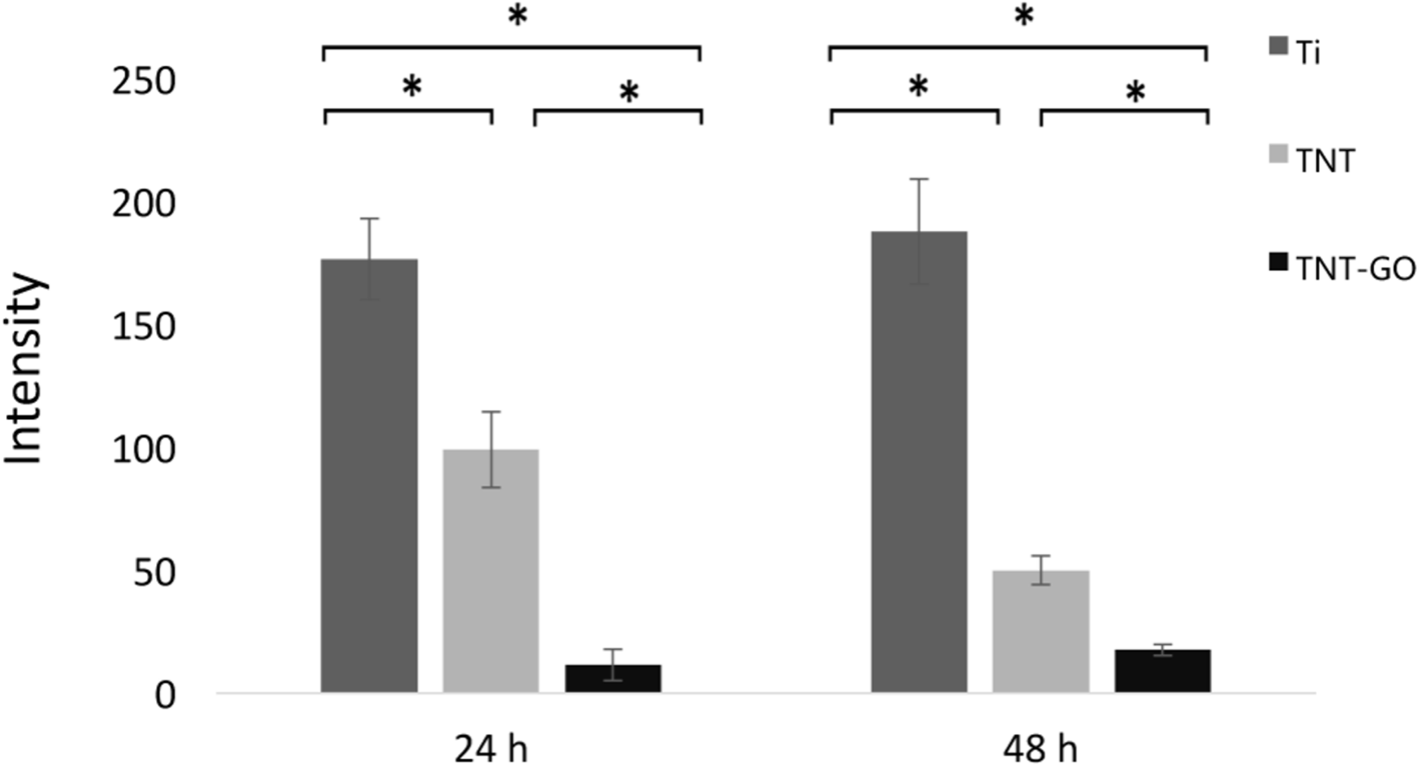
Results of quantitative analysis of ROS production under LPS stimulation at 24 and 48 h. *p < 0.05

The ROS generation on the surface of the RAW 264.7 samples stimulated by LPS was detected with a ROS kit. The highest expression of ROS was observed in the Ti group, followed by the TNT group (Fig. 9) and finally the TNT–GO group. The fluorescence intensity of each group was quantitatively analysed (Fig. 10); at 24 and 48 h, the expression of ROS between the three groups was significantly different, with significantly few ROS in the TNT–GO group.

## Discussion

Understanding the inflammatory response of a biomaterial after implantation can help in designing materials that can modulate macrophage polarization into an anti-inflammatory pro-healing phenotype (M2) that induces a favourable immune response after implantation. Several studies have shown that the surface roughness, surface morphology, charge, chemical composition, wettability, and biological factors of the material [29–33] affect the polarization of macrophages and the activation and production of pro-inflammatory factors in macrophages [34, 35].

The results of this study revealed that the nano-morphology had a favourable regulatory effect on the polarization of macrophages and reduced the level of inflammation. A previous study demonstrated that a highly ordered nano-morphology significantly impacted the shape and phenotype of macrophages [6]. Notably, TNTs with a diameter of 100 nm could provide more protein adsorption sites because the gap between the nanotubes can serve as a nutrient supply pathway [36]. Moreover, the nanotube structure itself can reduce the inflammatory response [37]. In addition, changes in the implant surface roughness and morphology may indirectly impact macrophage functions, including survival, adhesion, gene expression, and secretion [38]. In addition, previous studies have shown that nanoscale surfaces are more conducive to inflammation regulation and osteogenic gene expression than microscale surfaces, and the introduction of surface microgrooves, micropillars and other structures significantly affects macrophage adhesion, spreading, cytoskeletal remodelling and transcriptome profile [6, 39, 40]. The results of this study show that nanotubes with a diameter of approximately 100 nm have good roughness and can attenuate inflammatory responses. Nanotubes significantly enhanced the hydrophilicity of titanium, and after loading GO, the hydrophilicity was further improved.

Studies have shown that the surface wettability of solid materials is mainly determined by the chemical composition, surface structure, and surface energy of the materials [41]. Changes in roughness will affect the water contact angle on the material surface. When the intrinsic contact angle < 90°, the greater the roughness, the stronger the hydrophilicity [42]. In this study, Ti had the lowest roughness and the largest water contact angle. After the TNT surface structure was formed by anodisation, the water contact angle of the material was significantly reduced, forming a hydrophilic surface. Evidently, this nanostructure could increase the specific surface area and roughness of materials, which in turn strengthened hydrophilicity. In addition, the TNT–GO group had higher hydrophilicity than the TNT group, which was due to the abundant oxygen-containing functional groups on the surface of GO. These functional groups can form hydrogen bonds with water molecules and improve the hydrophilicity of the material.

It is generally believed that activated macrophages expand rapidly and extensively. The pro-inflammatory microenvironment is conducive to the activation of macrophages and activates their proliferation ability, while the anti-inflammatory microenvironment weakens their proliferation rate [43]. The TNT-GO in this study has a unique surface morphology and better hydrophilicity, forming an anti-inflammatory microenvironment and inducing the polarization of macrophages toward M2, thus inhibiting the proliferation and adhesion capabilities of macrophages.

Recent studies have shown that the surfaces of hydrophilic biomaterials can promote macrophage apoptosis, increase the secretion of anti-inflammatory cytokines, and reduce the levels of pro-inflammatory factors [44, 45]. With an increase in the surface roughness, hydrophilicity, and protein adsorbability of biomaterials, macrophages may interact with adsorbable proteins and promote the M2 polarization of macrophages. These results are consistent with those of previous studies focused on the relationship between morphology and macrophage polarization. Notably, a stretched morphology indicates that the cells are activated and can migrate, whereas a round morphology indicates that the cells are not activated and are immobile [13, 46]. The SEM images (Fig. 4) revealed obvious stretching in the Ti group cells after LPS stimulation, in contrast with the round macrophages on the surface of the TNT–GO. Taken together, the CCK8, cell fluorescence staining, and ELISA results revealed that the proliferation and activation of macrophages as well as the level of inflammation in the TNT and TNT–GO groups were lower than those in the Ti group. The expression of M2-related cytokines and proteins in the TNT–GO group was higher than that in the other two groups. Compared with macrophages cultured on pure Ti surface, those on the TNT–GO composite material exhibited a lower secretion of pro-inflammatory cytokines IL-6 and TNF-α. However, TNT–GO promoted the secretion of M2-related factor IL-10. The expression of the M2-related proteins CD163 and ARG was also significantly increased on TNT–GO. Previous studies have shown that TNF-α is a cytokine involved in systemic inflammation and acute phase responses, while IL-10 plays a major role in tissue remodelling and inhibiting inflammatory immune responses [34, 47, 48]. The production of IL-10 can enhance the positive feedback of M2 macrophages [49] and facilitate the vascularisation of biomaterials during tissue remodelling, thus improving the overall properties of the biomaterials to achieve their intended functions.

Therefore, the TNT–GO material with a nano-morphology and good hydrophilicity was more conducive to mitigating the inflammatory response of macrophages, facilitating the M2 polarization of macrophages and thus promoting tissue healing and repair [50, 51].

In addition, the GO coating was negatively charged and provided a large number of wrinkles on the material surface, which changed the surface morphology and may have affected cellular reactions such as diffusion [52]. High hydrophilicity and nano-morphology are favourable factors for the M2 polarization of macrophages. The designed TNT–GO material can induce M2 polarization, thereby promoting subsequent tissue healing.

The inflammatory response was more effectively reduced as well as the expression of anti-inflammatory factors and proteins and the M2 polarization of macrophages were promote on the TNT–GO surface in relation to those on the TNT surface. These results indicate that the GO coating itself also played an anti-inflammatory role.

To explore the molecular mechanism of TNT–GO-mediated immune regulation, this study used transcriptome sequencing to detect macrophage gene expression profiles. Gene ontology and KEGG enrichment analyses showed that many DEGs existed in the extracellular space. Oxygen binding, peroxidase activity, NADH dehydrogenase activity, nitrogen metabolism, extracellular matrix structural constituents, and calcium ion binding are closely associated with the ROS, including oxygen-containing free radicals and peroxides that can easily form free radicals related to oxygen metabolism in organisms. ROS is a natural by-product of normal oxygen metabolism and plays an important role in cell signalling and homeostasis [53]. However, in inflammation, the ROS levels increase sharply, seriously damaging the cell structure. Excessive and uncontrolled ROS are important factors in inflammation-induced tissue damage [53, 54], and ROS have been confirmed to be a pro-inflammatory factor in acute kidney injury and tubulointerstitial nephritis [55]. The oxygen free radicals in the cell mainly originate from the mitochondria; complexes I (NADH dehydrogenase) and III (Coenzyme Q-cytochrome C reductase) in the electron transport chain are the main sites of oxygen free radical production. Jiang et al. [56] showed that reduced NADH dehydrogenase activity could inhibit mitochondrial complex-I-mediated ROS outbreaks. Both active nitrogen and ROS are key factors affecting oxidative stress, and they also influence each other. Active nitrogen can regulate the activity of key enzymes involved in ROS homeostasis [57, 58]. In addition, studies have shown that active oxygen components such as oxygen free radicals damage the DNA structure, oxidise sulfhydryl groups in proteins, and degrade extracellular matrix components through lipid peroxidation [59]. The transcriptome sequencing results of this study showed that the reduction of NADH dehydrogenase activity, NO levels, and ROS production occurred on the TNT–GO surface, in addition to the change in the free radical microenvironment.

Recent studies have also demonstrated that microenvironmental factors such as free radicals can affect macrophage polarization [60]. Han et al. [61] confirmed the regulatory effect of dispersed GO on macrophage polarization and its application in myocardial infarction. The results of this study revealed that GO can be used as an antioxidant to reduce inflammation and the inflammatory polarization of macrophages by reducing intracellular ROS and downregulating the polarization of M1 macrophages and the secretion of related inflammatory cytokines, thereby reducing the inflammatory response. GO can also be used as a carrier of interleukin-4 plasmid DNA (IL-4 pDNA). Chen et al. [62] found that targeting metabolic reprogramming with LDHA reduced ROS generation and induced the polarization of M2 macrophages. In addition, several studies [63, 64] have shown that GO can protect cells from ROS-mediated death and reduce the damage caused by ROS, thereby improving therapeutic effects.

Moreover, this study demonstrated that a GO coating, similar to dispersed GO, significantly inhibited ROS production (Figs. 9 and 10) and regulated the M2 polarization of macrophages. The secretion of pro-inflammatory factors TNF-α and IL-6 was reduced and that of anti-inflammatory factor IL-10 was promoted on the TNT–GO surface, thus creating a good microenvironment for tissue healing. Therefore, modifying the surface of TNT with GO can inhibit the production of ROS, affect macrophage polarization, and regulate M2 polarization, all of which are highly desirable in clinical applications. This study examined the effect of GO on macrophage gene expression through transcriptome sequencing. The results showed that a large number of DEGs clustered in functions such as oxygen binding, peroxidase activity, NADH dehydrogenase activity, nitrogen metabolism, extracellular matrix structural components, and calcium ion binding, and these functions are closely related to ROS. The results of subsequent experiments confirmed that GO can inhibit the generation of ROS. Therefore, it is speculated that GO may regulate ROS production by affecting NADH dehydrogenase activity, nitrogen metabolism, extracellular matrix structural components, calcium ion binding, and other functions of macrophages, ultimately affecting the expression of inflammatory factors; however, more experiments are required to explore the exact mechanisms in further detail.

Overall, the results of this study suggest that the anti-inflammatory effects of TNT–GO may be related to its nano-morphology, good hydrophilicity, and decreased ROS production. In addition, the M2 polarization of macrophages was more effectively induced and the LPS-stimulated inflammatory response was weakened on the TNT–GO surface in relation to those on the TNT surface, which may be attributed to the loading of GO. Furthermore, ROS production was more effectively decreased and changes in the microenvironment, such as free radicals, were induced on the TNT–GO surface.

## Conclusions

In this study, GO was electrodeposited onto the surface of anodised TNT, and the TNT array and GO were found to regulate the inflammatory response of the RAW264.7 cells on the Ti surface. The inflammatory response and expression of pro-inflammatory factors were reduced and the M2 polarization of macrophages and secretion of anti-inflammatory factors were promoted on the surface of single-layer TNT–GO material, which may be attributed to the reduced production of intracellular ROS. Therefore, TNT–GO is a promising material with immunoregulatory capabilities that can promote faster, more effective tissue healing. However, more in vivo experiments are needed to verify the applicability of TNT–GO.

### Supplementary Information

Supplementary Material 1.

Supplementary Material 2.

Supplementary Material 3.

Supplementary Material 4.

Supplementary Material 5.

Supplementary Material 6.

Supplementary Material 7.

Supplementary Material 8.

Supplementary Material 9.

## Data Availability

The datasets used and/or analysed during the current study are available from the corresponding author on reasonable request.

## Abbreviations

DEG: Differential Expression of Genes
GO: Graphene oxide
TNT: Titanium nanotubes
SEM: Scanning electron microscopy
ELISA: Enzyme–linked immunosorbent assay
